# Spectral Optical Readout of Rectangular–Miniature Hollow Glass Tubing for Refractive Index Sensing

**DOI:** 10.3390/s18020603

**Published:** 2018-02-16

**Authors:** Giulia Rigamonti, Valentina Bello, Sabina Merlo

**Affiliations:** Dipartimento di Ingegneria Industriale e dell’Informazione, Università di Pavia, 27100 Pavia, Italy; giulia.rigamonti01@universitadipavia.it (G.R.); valentina.bello01@universitadipavia.it (V.B.)

**Keywords:** rectangular glass tubing, optical resonator, refractive index sensing, optical spectral measurements, response curves

## Abstract

For answering the growing demand of innovative micro-fluidic devices able to measure the refractive index of samples in extremely low volumes, this paper presents an overview of the performances of a micro-opto-fluidic sensing platform that employs rectangular, miniature hollow glass tubings. The operating principle is described by showing the analytical model of the tubing, obtained as superposition of different optical cavities, and the optical readout method based on spectral reflectivity detection. We have analyzed, in particular, the theoretical and experimental optical features of rectangular tubings with asymmetrical geometry, thus with channel depth larger than the thickness of the glass walls, though all of them in the range of a few tens of micrometers. The origins of the complex line-shape of the spectral response in reflection, due to the different cavities formed by the tubing flat walls and channel, have been investigated using a Fourier transform analysis. The implemented instrumental configuration, based on standard telecom fiberoptic components and a semiconductor broadband optical source emitting in the near infrared wavelength region centered at 1.55 µm, has allowed acquisition of reflectivity spectra for experimental verification of the expected theoretical behavior. We have achieved detection of refractive index variations related to the change of concentration of glucose-water solutions flowing through the tubing by monitoring the spectral shift of the optical resonances.

## 1. Introduction

In recent years, increasing attention has been given to the realization of label-free optical sensors able to measure the refractive index (RI) of samples [1,2,3,4]. Label-free sensing has become of great interest, since, with respect to techniques that make use of exogenous markers, it is safer, usually cheaper and it does not contaminate the sample under test. Furthermore, optical readout allows remote sensing of the investigated parameters, being thus minimally invasive, an issue that must be carefully taken into account when dealing with biological and biomedical analyses.

Several types of sensors that fulfil the mentioned requirements have been investigated, such as resonant micro-cavities [2,5,6,7,8], photonic crystals [9,10,11,12,13,14], ring resonators [15,16,17] and fiber-optic sensors, such as Fiber Bragg Gratings (FBG) and Long Period Gratings (LPG) [4,18,19,20], in general combined with optical spectral readout. Many architectures have been developed and tested: in [21], for instance, Li et al. proposed a fiber-optic Fabry–Perot interferometer (FPI) based on a single-mode fiber ending with an open microcavity with the inner surfaces covered with gold films. With this design, they obtained a minimum detectable RI step of the order of 10^−6^ RIU. Wang et al., in [22], proposed a RI sensor based on a 2D photonic quasicrystal structure composed of silicon rods, obtaining a sensing accuracy of 10^−4^ RIU. 3D structures have been as well extensively investigated: Wu et al., for example, developed a 3D polymeric photonic crystal RI sensor fabricated by femtosecond laser writing, and detected RI changes as low as 6 × 10^−3^ RIU [14]. 

Kim et al. [15] exploited vertically coupled micro-ring resonator in polymeric waveguides, obtaining detection limit of the order of 10^−6^ RIU. Coupling a portion of a capillary fiber into a microfiber immersed in cured low-refractive index polymer, Sumetsky et al. [23] reached sensitivities around 800 nm/RIU by monitoring the transmitted spectrum as solutions at increasing RI were flowing into the capillary channel. In [17] a round capillary coupled with a tapered fiber was investigated, and label free RI detection was performed observing changes as low as 2.8 × 10^−7^ RIU. Liu et al. [24] proposed a FPI cavity fabricated into an SMF with two open faces by femtosecond-laser induced water-breakdown and obtained a sensitivity S = 1147.48 nm/RIU and, considering the resolution (0.01 nm) of the spectrometer, calculated a detection limit equal to 1.29 × 10^-4^ RIU.

Chryssis et al. [19] proposed an FBG sensor developed by etching part of the core of the fiber and achieved a sensitivity of 1394 nm/RIU by monitoring the RI change of surrounding media. By considering a spectral resolution of 0.01 nm, they estimated a minimum detectable RI step of 7.2 × 10^−6^ RIU. In [18], Sun et al. demonstrated the functionality of a hybrid sensor composed by an LPG and a chirped fiber Bragg grating (CFBG), obtaining a best RI resolution of about 5.6 × 10^−6^ RIU. Baldini et al. [25] proposed a fiber tip sensor embedded with FBG-LPG for monitoring RI of different solutions with an accuracy of about 4 × 10^−3^ RIU. 

Despite the achieved results, there is still the need of demonstrating the functionality of simple and low-cost devices that can be used as key elements of micro-opto-fluidic sensors matched with suitable readout methods. 

Rectangular–miniature hollow glass tubings [26,27,28,29] may represent an ideal solution to satisfy these necessities, being suitable for RI detection. These devices, commercially available in several formats, exhibit some interesting features. Their flat surface makes them particularly appropriate for non-invasive optical readout that does not modify or damage the sample. Furthermore, the rectangular cross-section strongly reduces the optical distortions and scattering typical of round cross-section capillaries [30].

Rectangular capillaries already find wide application in the field of acustophoresis, overcoming the limit of round capillaries. In [27], for example, Hammarstrom et al. employed rectangular micro-tubings as resonators for acoustic standing wave trapping, and demonstrated their application for aspirating, trapping and dispensing red blood cells. Evander et al. [31] demonstrated the use of rectangular capillaries as core elements of a micro-fluidic system for the non-contact trapping of particles by means of acoustophoresis technology. In [30], rectangular tubings were employed for capillary zone electrophoresis, and demonstrated great efficiency in heat dissipation compared to standard circular capillaries.

As core elements of refractometric sensors, rectangular tubings present the additional advantage of allowing to perform the RI detection on extremely small volumes of fluids and without the need of labeling substances, an issue that must be taken into account when dealing with biological samples [32]. 

In a previous work [26], we reported preliminary results relative to the theoretical and experimental behavior of glass capillaries with rectangular cross section exhibiting a symmetrical geometry, thus with channel depth and walls thickness of the same size; in particular, two kinds of devices were investigated as RI sensors with characteristic dimensions of 50 µm and 20 µm. Proof of principle of RI sensors based on these devices was demonstrated by exploiting the optical readout method based on spectral reflectivity detection, in direction orthogonal to the flat side, and correlating the variations in the spectral line-shape with the RI of the filling solution.

Here, we analyse the theoretical and experimental optical features of rectangular tubings with asymmetrical geometry, now commercially available; in particular, we have considered devices characterized by a channel depth of 30 µm and glass walls thickness of 21 µm as well as by a channel depth of 50 µm and glass walls thickness of 35 μm. Initially, a deeper insight is given to the origins of the complex line-shape of the response in the near-infrared wavelength domain, still considering the previously exploited optical readout method based on spectral reflectivity measurements. In particular, a Fourier transform analysis is conducted for a better understanding of the contribution of each cavity formed by the tubing flat walls and channel. Moreover, we investigate the impact on the RI sensitivity of a tubing structure with walls thinner than the channel. Given the same channel depth, tubings with asymmetrical geometry characterized by thinner walls, with respect to standard symmetrical devices, allow to achieve higher sensitivity values. This result, theoretically demonstrated and experimentally verified, may be intuitively explained as a consequence of the lower contribution of the thinner glass walls, clearly insensitive to RI fluid variations, on the total optical thickness crossed by the readout radiation.

In this work, an instrumental configuration, based on standard telecom fiberoptic components and a semiconductor broadband optical source emitting in the near infrared wavelength region centered at 1.55 µm, is then used for the acquisition of reflectivity spectra and experimental verification of the expected theoretical behavior. We demonstrate the effect of refractive index variations, induced by changes of concentration of glucose-water solutions flowing through the tubing, on the wavelength position of the optical resonances observed on the detected spectra.

## 2. Structure and Theoretical Optical Features of Rectangular–Miniature Hollow Glass Tubings

**Figure 1:**
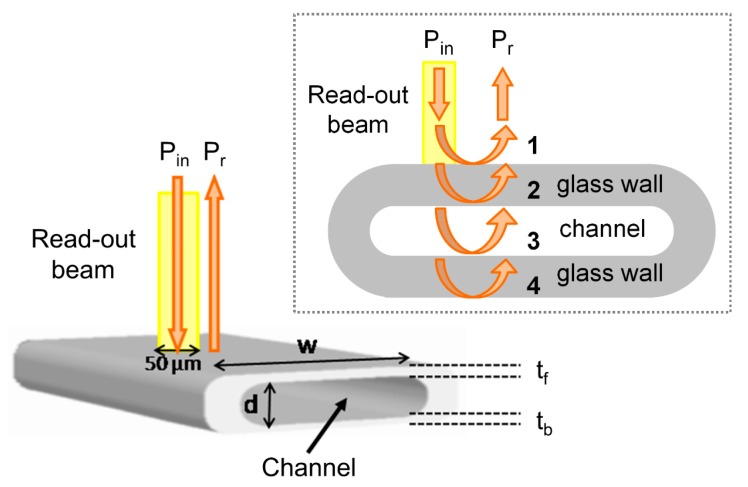
Schematic structure of the rectangular–miniature hollow glass tubing, with input illuminating beam P_in_ and back reflected beam P_r_. d: channel depth, w: side width, t_f_: thickness of front wall; t_b_: thickness of back wall. The inset shows a schema of a capillary longitudinal cross-section, where the four capillary interfaces are highlighted.

Rectangular–miniature hollow glass tubings (Vitrotubes™, VitroCom, Mountain Lakes, NJ, USA) with channel depth (d) and glass wall thicknesses (t_f_ and t_b_) of a few tens of micrometers can be modeled as a sequence of three layers of different materials limited by four, flat dielectric interfaces: air-glass, glass-channel, channel-glass and glass-air, respectively numbered from 1 to 4 in Figure 1. When they are illuminated by broadband electromagnetic radiation perpendicularly to the channel axis and to the surface of the wider flat side (Figure 1), back-reflected radiation shows a wavelength spectrum with a complex line-shape due to interference effects among the fields reflected at the refractive index (RI) discontinuities. The field reflection coefficient of each interface can be easily calculated by means of Fresnel equations [33] and the overall reflectance can be obtained by cascading the effects of various Fabry-Perot etalons, thus keeping into account multiple reflections.

**Figure 2:**
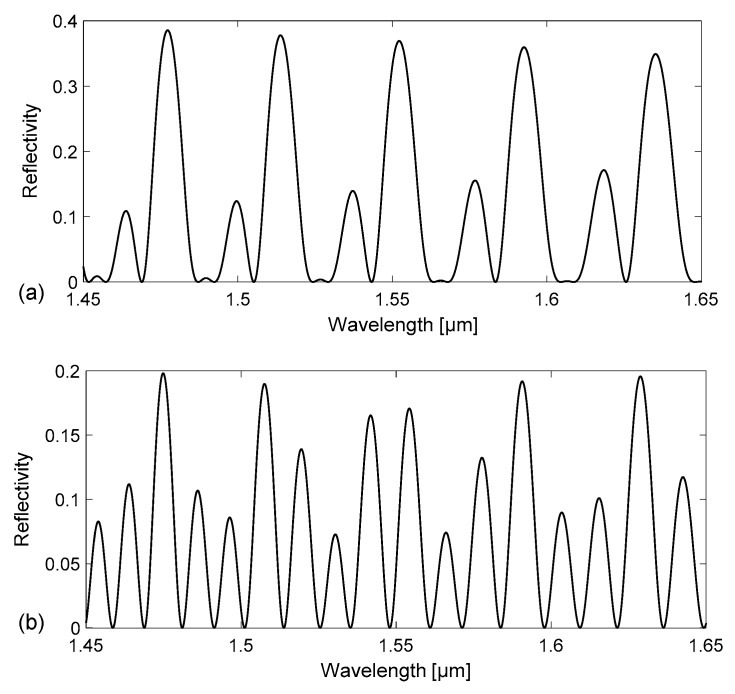
Calculated reflectivity spectra R(λ) = P_r_(λ)/P_in_(λ) for a rectangular–miniature hollow glass tubing with d = 30 µm, t_f_ = t_b_ = 21 μm (nominal dimensions): (**a**) empty channel; (**b**) channel filled with water.

**Figure 3:**
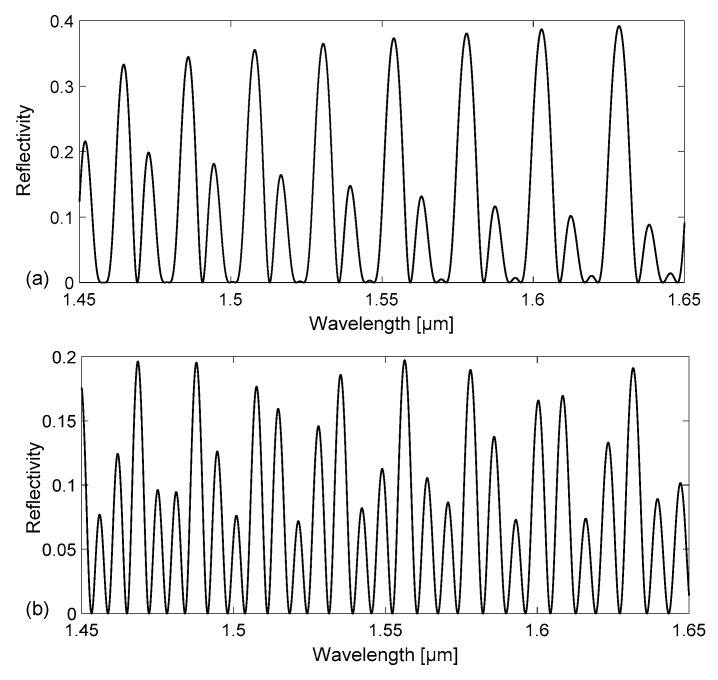
Calculated reflectivity spectra R(λ) = P_r_(λ)/P_in_(λ) for a rectangular – miniature hollow glass tubing with d = 50 µm, t_f_ = t_b_ = 35 μm (nominal dimensions): (**a**) empty channel; (**b**) channel filled with water.

Thus, the device reflectance strongly depends on the refractive index of the channel filling. Using this simple model, we have carried out numerical simulations to calculate the spectral reflectivity of a tubing with channel depth d = 30 µm, side width w = 300 µm, and t_f_ = t_b_ = 21 μm (nominal dimensions of Part#5003 by VitroCom) and of a tubing with d=50 µm, w = 500 µm, and t_f_ = t_b_ = 35 μm (nominal dimensions of Part#5005 by VitroCom) in the wavelength range 1.45–1.65 μm. The calculated reflectivity spectra R(λ) = P_r_(λ)/P_in_(λ), where P_r_ is the reflected and P_in_ the incident optical power density, are reported in Figure 2 and Figure 3, for the smaller and larger tubing, respectively. While Figure 2a and Figure 3a refer to the case of an empty device, Figure 2b and Figure 3b are obtained by considering the channel filled with water. 

Although all spectra show peaks and valleys, corresponding to resonance wavelengths of the device, they exhibit very different line-shapes. Peaks with higher amplitude are observed, for both tubing dimensions, in the spectra relative to the empty device, due to the higher refractive index step at the interfaces between wall and air-filled channel. More peaks are counted in the same wavelength interval on the spectrum relative to the water-filled tubing compared to what is observed for an empty tubing with the same channel depth. Comparing the results attained for tubings of different sizes, more peaks are counted in the same wavelength interval in the spectrum relative to a tubing with larger dimensions, but same filling.

**Figure 4:**
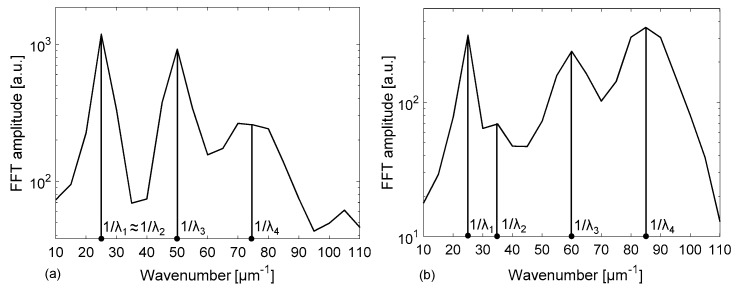
Results of the Fast Fourier Transform applied to the calculated spectral reflectivity reported in Figure 2 for a rectangular – miniature hollow glass tubing with d = 30 µm, t_f_ = t_b_ = 21 μm (nominal dimensions): (**a**) empty channel; (**b**) channel filled with water.

**Figure 5:**
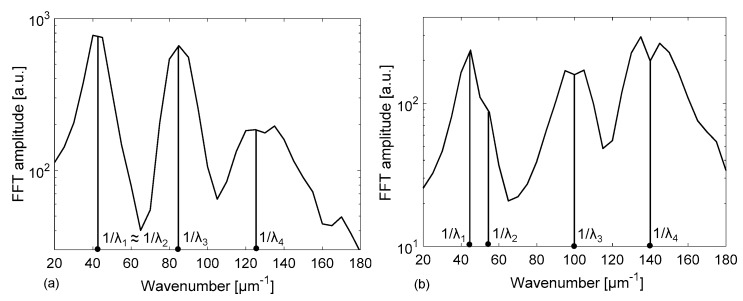
Results of the Fast Fourier Transform applied to the calculated spectral reflectivity reported in Figure 3 for a rectangular–miniature hollow glass tubing with d = 50 µm, t_f_ = t_b_ = 35 μm (nominal dimensions): (**a**) empty channel; (**b**) channel filled with water.

The origins of the complex line-shape of the spectral response due to the different cavities formed by the tubing flat walls and channel have been investigated using a Fast Fourier Transform (FFT) analysis, to identify the main Fourier components of the signal. Broad peaks are expected at different locations on the 1/λ horizontal (abscissa) axis, or wavenumber, corresponding to the inverse of the wavelength separation Δλ of each resonance, given by the relationship Δλ = λ^2^/(2 · *OT*) where *OT* is the optical path length of the considered cavity and λ is the wavelength position of the resonance (for example, a reflectivity minima). For the cavity corresponding to the channel (between interfaces 2–3, see Figure 1), Δλ_channel_ is therefore estimated by placing *OT*= *OT*_channel_ = n_f_ · d, where n_f_ is the filling fluid RI. For the cavity corresponding to the whole device (between interfaces 1–4), Δλ_device_ is calculated using *OT* = *OT*_device_ = n_f_ · d + n_glass_ · (t_f_ + t_b_). Analogously, for the cavity corresponding to a single wall (interfaces 1–2 and 3–4), we have to consider *OT*_wall_ = n_glass_ · t_f_ and for the cavity formed by a wall plus the channel *OT*_w+c_ = n_glass_ · t_f_ + n_f_ · d. For increasing values of 1/λ, the first peak is expected at 1/λ_1_ = 1/ Δλ_wall_, a second one at 1/λ_2_ = 1/Δλ_channel_, then the third at 1/λ_3_ = 1/Δλ_w+c_ and another around 1/λ_4_ = 1/Δλ_device_. The FFTs of the reflectivity spectra previously reported in Figure 2 and Figure 3 are shown in Figure 4 and Figure 5, respectively. By concentrating our attention on Figure 4a, relative to an empty tubing with channel depth d = 30 µm, we can actually distinguish only three peaks, since 1/Δλ_channel_ ≈ 1/Δλ_wall_ ≈ 25 µm^−1^, and thus 1/λ_1_ ≈ 1/λ_2_. Moving our attention to the graph in Figure 4b, four peaks become now evident since 1/λ_2_ = 1/Δλ_channel_ > 1/Δλ_wall_ = 1/λ_1_, due to the contribution of the water refractive index that increases *OT*_channel_. Similarly, in Figure 5a, relative to an empty tubing with d = 50 µm, we can distinguish three peaks, since 1/Δλ_channel_ ≈ 1/Δλ_wall_ ≈ 42 µm^−1^, and thus 1/λ_1_ ≈ 1/λ_2_ whereas in Figure 5b we can separate the contribution at 1/λ_1_ from that at 1/λ_2_, since 1/λ_2_ = 1/Δλ_channel_ is slightly higher than 1/λ_1_ = 1/Δλ_wall_ due to the contribution of the water refractive index.

Table 1 summarizes the theoretically calculated values of *OT*, Δλ and 1/λ for both tubing dimensions (nominal values), with and without water filling.

**Figure 6:**
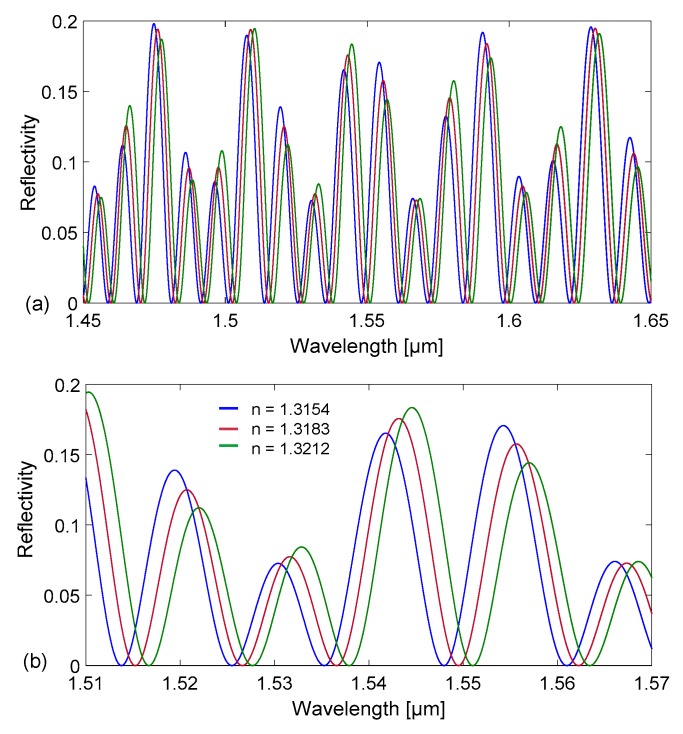
Calculated reflectivity spectra R(λ) = P_r_(λ)/P_in_(λ) for a rectangular–miniature hollow glass tubing with d = 30 µm,t_f_ = t_b_ = 21 μm (nominal dimensions) considering three different values of refractive index for the filling fluid: (**a**) spectra in the wavelength range 1.45–1.65 µm; (**b**) zoom in the range 1.51–1.57 µm.

As already mentioned, when comparing Figure 2a with Figure 2b, as well as Figure 3a with Figure 3b, it becomes evident that the profile of the spectral reflectivity does severely change when a fluid flows through the capillary. To investigate the potentiality of these devices as fluid RI sensors, we have carried out numerical simulations to calculate the spectral reflectivity of the tubing filled by fluids with increasing values of refractive index, with respect to water, considered as the reference fluid. As an example, Figure 6 illustrates reflectivity spectra, calculated for the tubing with d = 30 µm, considering three different values of refractive index for the filling fluid. In Figure 6a, data are reported for the wavelength range 1.45–1.65 µm as in Figure 2 and Figure 3 whereas Figure 6b is the zoom in the narrower range 1.51–1.57 µm, that corresponds to the bandwidth of the optical source employed in the experimental tests. It can be noticed that the wavelength position of the minima shifts towards longer wavelength when the refractive index of the fluid increases. In principle, if the channel depth were much smaller than the wall thickness so that *OT*_channel_ would remain substantially different from *OT*_wall_ even with fluid filling, as in [34], the shift could be recovered from the Fourier transform after identification of the channel mode. In the case of the rectangular hollow tubing employed in this work, with *OT*_wall_ of the same order of magnitude of *OT*_channel_, the reflectivity spectrum is the result of the superposition of all resonance modes and the shift as a function of the fluid refractive index is better detected directly in the wavelength domain. 

In order to investigate the theoretically expected performances of the employed devices as fluid RI sensors, we further performed numerical simulations in the wavelength range of 1.51–1.57 µm for a wider interval of RI values. In particular, we considered RIs in the range from 1.3154 up to 1.3386 RIU, corresponding to glucose concentrations in water between 0 and 16% (separated by a step of 1%) filling the tubing with d = 30 µm, and RI values in the range of 1.3154–1.3299 RIU, corresponding to glucose concentrations from 0 to 10%, for the tubing with d = 50 µm. The RI interval was chosen in order to investigate the widest exploitable RI interval, depending on the distance between two consecutive minima (or maxima) of the same spectrum, that limits the maximum RI variation detectable without ambiguity. By reporting the detected position in terms of wavelength of the spectral minima as a function of the relative considered RI value, we obtained the theoretical response curves for both formats of glass tubings. Defining the sensitivity as S = dλ_min_/dn, the theoretical sensitivities were retrieved as the slope of the device response curves. Comparable values were found for both devices. In particular, the numerical evaluation of the device performances led to a maximum sensitivity value S_max_ = 500.03 nm/RIU for the tubing with d = 30 µm, and to S_max_ = 507.5 nm/RIU for the tubing with d = 50 µm. As mentioned before, the reflected spectra result from the superimposition of contributes coming from different cavities, but where just the cavities incorporating the channel are affected by the fluid RI. A device is expected to be more sensitive as the weight of the channel on its overall thickness is higher. For the nominal devices we considered, i.e. the one characterized by t_f_ = t_b_= 35 µm and d = 50 µm and the one with t_f_ = t_b_ = 21 µm and d = 30 µm, the impact of the channel depth on the total optical thickness is the same, thus similar RI sensing performances should be achieved. 

## 3. Experimental Results

### 3.1. Instrumental Configuration

**Figure 7:**
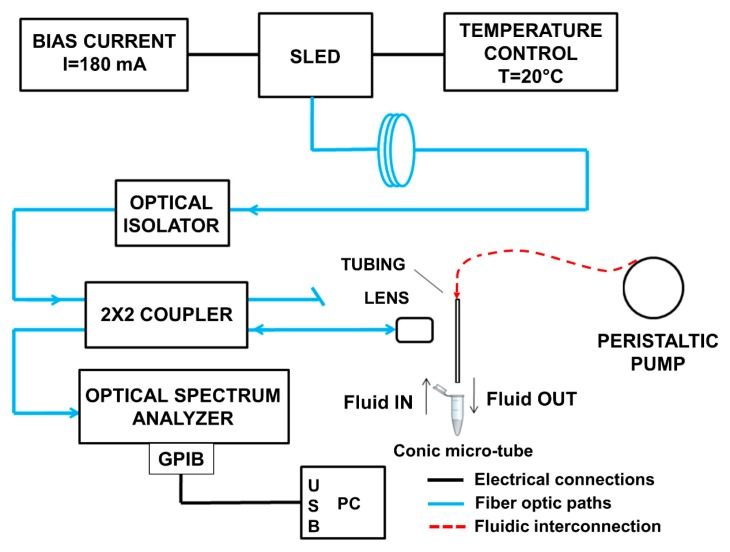
Block diagram of the setup applied in the experiments. The different kinds of connections (electrical, optical and fluidic) are highlighted. SLED: Superluminescent Light Emitting Diode.

Experimental verification of the expected theoretical behavior has been carried out thanks to an all-fiber setup shown in Figure 7. A fiber-pigtailed Superluminescent Light Emitting Diode (SLED, Exalos EXS1510-2111) with Gaussian emission profile centered at λ = 1.549 μm has been employed for illuminating the tubing. The optical power coupled in standard single-mode optical fibers is approximately 1.8 mW, when the SLED is driven by a pumping current I = 180 mA, at a temperature of 20 °C.

The fiber-coupled emitted radiation is guided through an optical isolator, to avoid undesired back reflections into the SLED, and then through a 2 × 2, 50:50 fiber-coupler. One of the output ports of the coupler ends with a micro-lens that shines the beam orthogonally onto the flat side of the rectangular tubing. The other output port is terminated with an angled connector to eliminate its contribution in reflection. Back reflected light is coupled back in the optical fiber and redirected to an Optical Spectrum Analyzer (OSA, Agilent 86142B, Agilent Technologies, Santa Clara, CA, USA) that allows to visualize the power density spectrum of the reflected light as a function of the wavelength. The spectrum analyzer is connected to a computer for data acquisition. The tubing is mounted on a manual micro-positioner that can be moved in the x, y and z direction, in order to maintain it in a stable and fixed position that optimizes back coupling. The liquid samples, stored in conic micro-tubes, flow into the channel by capillarity, without any external aids and exit by turning on a peristaltic pump.

### 3.2. Experimental Spectra

To confirm the expected theoretical results, we proceeded with the experimental testing of one kind of asymmetrical rectangular glass tubing as RI sensor. Given the same expected behavior in terms of sensitivity for both kind of devices, we choose to test the tubing with the narrower channel, i.e. the one with d = 30 µm, thus allowing to work with smaller quantities of sample. 

**Figure 8:**
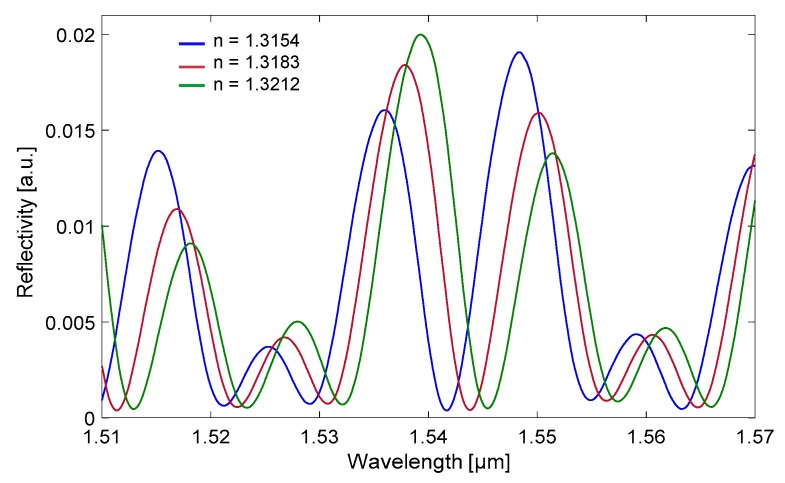
Experimental spectra of the reflected optical power, normalized to the input power spectrum, measured on a rectangular – miniature hollow glass tubing with d = 30 µm, t_f_ = t_b_ = 21 μm (nominal dimensions) filled with glucose-water solutions at three different glucose concentrations C in %(weight/volume). Blue trace: C = 0% [W/V] corresponding to RI = 1.3154, red trace: C = 2% [W/V] corresponding to RI = 1.3183, green trace: C = 4% [W/V] corresponding to RI = 1.3212 (all RI values are calculated as in [35,36]).

Figure 8 reports the reflected power spectra collected on a tubing with channel depth d = 30 µm, side width w = 300 µm, and t_f_ = t_b_ = 21 μm (nominal dimensions of Part#5003) by flowing glucose-water solutions at increasing concentrations (and thus RI). The wavelength range 1.51–1.57 µm corresponds to the emission bandwidth of the SLED. The spectral shift of minima or maxima is, as expected, toward longer wavelength for increasing values of RI. The behavior is quite similar to what was found theoretically, in particular with regard to the number of peaks and valley in the same wavelength range and the shift magnitude. The differences in the line shape between the experimental results in Figure 8 and the numerical results in Figure 6b can be attributed to the tolerances in the real tubing dimensions (10% channel depth and 20% wall thickness), which affects the absolute position of maxima and minima, and to coupling losses due to material diffusion, which affects the amplitude.

**Figure 9:**
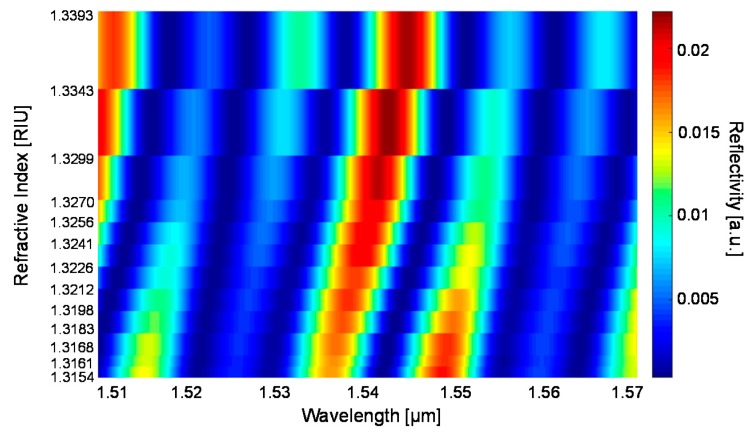
2D view of a 3D reconstruction of the sequence of experimental spectra measured on a rectangular–miniature hollow glass tubing with d = 30 µm, t_f_ = t_b_ = 21 μm (nominal dimensions) filled with glucose-water solutions in 13 different glucose concentrations and thus 13 different RI values. Amplitude values are represented using false colours, indicated on the right side of the graph.

Data have been actually collected on the same tubing using glucose-water solutions at 13 different concentrations and the results are reported in Figure 9 as 2D view of a 3D reconstruction of the sequence of spectra. Amplitude values are represented using false colours, indicated on the right side of the graph.

**Figure 10:**
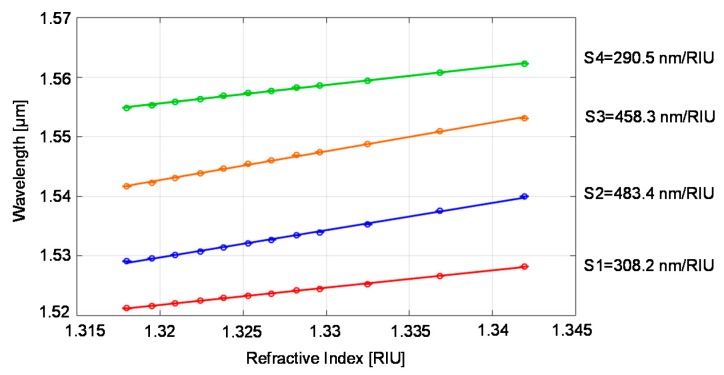
Wavelength positions of R minima as a function of glucose solution RI: mean values (empty circles) and standard deviations (error bars) calculated on three spectral acquisitions on a rectangular tubing with d = 30 µm, t_f_ = t_b_ = 21 μm (nominal dimensions). Straight lines represent the best linear fitting of the data.

Monitoring the spectral response allows estimation of RI variations of solutions flowing into the tubing channel with respect to a reference fluid, due for example to a change of concentration of the solute. Toward this aim, the response curves reported in Figure 10 have been obtained by fitting the wavelength position of the four minima of the spectra in the range 1.51–1.57 μm as a function of the refractive index. Defining the sensitivity as S = dλ_min_/dn (that is the slope of the response curves), values in the range of 290.1−484.5 nm/RIU have been found. These values are in good accordance with the numerical ones and higher than those experimentally obtained previously on symmetrical tubings [26]. Moreover, they are comparable with sensitivity values found in literature, but often obtained with devices fabricated or custom modified by means of sophisticated micromachining techniques. Considering the experimentally obtained standard deviations and sensitivities, we estimated a resolution, or, as indicated by other authors, LoD, in the range of 10^−4^–10^−5^ RIU, through the equation 3Ϭ/S, where Ϭ is the experimental standard deviation. Eventually, retrieving the RI of solutions with known solute and solvent allows estimating the concentration. The best sensitivity value found in the experimental verification was slightly lower than that expected theoretically: probably, this result can be explained by a mismatch between the nominal and effective geometrical dimensions of the device. To estimate the actual dimensions, we characterized the tubing employed for refractometric detection through low-coherence interferometric measurements, that give the optical thickness as *OT*_g_ = n_g_ · x, where n_g_ is the group refractive index of the material and x its geometrical thickness, as explained in [37,38]. We thus found the device geometrical dimensions by placing n_g,glass_ = 1.4752 RIU [39] and n_g,air_ = 1 RIU, obtaining t_f_ = 22.40 µm t_d_ = 22.30 µm and d = 29.84 µm. The difference between the nominal values and the actual ones, though compatible with the given tolerances, may explain the minor discrepancy between the experimental and theoretical highest sensitivities. 

## 4. Conclusions

The operating principle of the micro-opto-fluidic sensing platform that employs rectangular – miniature hollow glass tubings and an optical readout method based on spectral reflectivity detection has been described by defining an analytical model of the tubing that allows calculation of the output signal as superposition of the effects of different optical cavities. The Fourier transform analysis has demonstrated that the components of the complex line-shape of the spectral response are related to the different cavities formed by the tubing flat walls and channel. 

This effective and low-cost system provides performances comparable to those obtained with more expensive and more complex devices at the state of the art. The obtained results have been found in agreement with the theoretically expected values calculated through numerical simulations. In addition, the proposed sensor is suitable for the detection of a wide range of different biological fluids and samples. The use of read-out sources emitting in the near-infrared offers a double advantage, allowing to work in a wavelength region of minimum invasiveness for biological samples and to make use of standard optical components already commercially available as they have been developed for optical communications.

As future perspective, it would become very interesting to translate the spectral shift in amplitude variations at a single wavelength [40], for example using a laser and a photodiode for the detection of the reflected optical power, thus obtaining a compact, cheaper and potentially portable system.

## Figures and Tables

**Table 1:** Theoretical values of *OT*, Δλ and 1/λ for both tubing dimensions (nominal values), with and without H_2_O filling.

	Tubing 21-30-21			Tubing 35-50-35		
	*OT*[µm]	Δλ[nm]	1/λ[µm^−1^]	*OT*[µm]	Δλ[nm]	1/λ[µm^−1^]
Wall (1)	30.58	39.28	25.46	50.96	23.57	42.42
Channel (2)	30.00	40.05	24.97	50.00	24.03	41.62
Channel w H_2_O (2)	39.46	30.44	32.85	65.77	18.26	54.75
Wall+Channel (3)	60.58	19.80	50.50	100.96	11.86	84.31
Wall+Channel w H_2_O (3)	70.04	17.15	58.31	116.73	10.29	97.18
Whole device (4)	91.16	13.18	75.88	151.93	7.91	126.47
Whole device w H_2_O (4)	100.62	11.94	83.76	167.70	7.19	139.02
